# Human plasma fibronectin promotes proliferation and differentiation of odontoblast

**DOI:** 10.1590/1678-7757-2016-0442

**Published:** 2017

**Authors:** Jia TANG, Takashi SAITO

**Affiliations:** 1 Health Sciences University of Hokkaido, School of Dentistry, Department of Oral Rehabilitation, Division of Clinical Cariology and Endodontology, Hokkaido, Japan.

**Keywords:** Fibronectin, Odontoblasts, Cell differentiation, Cell proliferation

## Abstract

**Objective:**

To assess the effect of fibronectin (Fn) and porcine type I collagen (PCOL) on odontoblast-like cells *in vitro*.

**Material and Methods:**

Rat odontoblast-like cells (MDPC-23 cells) were inoculated and cultured on Fn-coated or type I collagen-coated substrates. Proliferation assay, alkaline phosphatase activity (ALP activity), mRNA expression of hard tissue-forming markers, and Alizarin red staining were investigated over a period of 10 days.

**Results:**

Cells maintained a high proliferation activity on Fn and PCOL even at a low seeding concentration (0.5×10^4^/mL) as demonstrated by CCK-8 assay. The proliferation activity of cells on Fn increases in a concentration-dependent manner while it reached a plateau after 10 µg/mL. Cells adopted long, thin and spindle shape on Fn(10-50) and PCOL. Parallel actin filaments were observed in MDPC-23 cells cultured on Fn and PCOL. ALP activity was markedly up-regulated on Fn and PCOL-coated surfaces. Importantly, gene expression of BSP (Fn10: 2.44±0.32; Fn20: 3.05±0.01; Fn30: 2.90±0.21; Fn40: 2.74±0.30; Fn50: 2.64±0.12; PCOL: 2.20±0.03) and OCN (Fn10: 2.52±0.23; Fn20: 2.28±0.24; Fn30: 2.34±0.21; Fn40: 2.34±0.25; Fn50: 2.20±0.22; PCOL: 1.56±0.16) was significantly enhanced on Fn and PCOL substrates as compared with control; moreover, expression of integrin beta 1 (ITGB1), an ubiquitous cell surface receptor was augmented in Fn(10-50) and PCOL groups simultaneously. In accordance with the ALP activity and gene expression data, calcific deposition in cells grown on Fn(10-50) and PCOL was observed as well.

**Conclusion:**

Despite the limitation of this study, the findings indicate that a surface coating of Fn enhances the proliferation, differentiation and mineralization of odontoblast-like cells by activation of integrin beta 1 (ITG B1). The promoting effects of Fn on MDPC-23 cells were achieved at a comparatively lower coating concentration than type I collagen (300 µg/mL). Specifically, it is suggested that the optimum coating concentration of Fn to be 10 µg/mL.

## Introduction

Fibronectin (Fn) is a dimeric multi-domain glycoprotein (about 450kDa per dimer) that is found in circulation or tissue extracellular matrix (ECM). Two major types of Fn are present in vertebrates arising from alternative splicing of its pre-mRNA: soluble plasma Fn (corresponds to the aforementioned CIg) and insoluble cellular Fn. Both of them contain various adhesive domains for cells and other proteins (collagen, fibrin, heparin), such as: the most widely known Arg-Gly-Asp (RGD) (αΙΙbβ3, αvβ3, αvβ6, αvβ1, α5β1, and α8β1 ligand)^9,21^, the synergic site of RGD: PHSRN^4,18^, Leu-Asp-Val (LDV) (α4β1 and α4β7 ligand)^9^, Arg-Glu-Asp-Val (REDV)^10^, IDAPS (α4β1 ligand)^15^, and KLDAPT (α4β1 and α4β7 ligand)^17^. The plasma form of Fn (pFn) is predominantly synthesized by hepatocytes, circulates in blood and deposits rapidly upon tissue injury to initiate hemostasis, this deposition process is independent of other hemostasis factors, such as fibrinogen, von Willebrand factor, β3 integrin, and platelet^30^. Cellular Fn (cFn) is mainly produced by fibroblast, to a lesser extent by other cells, such as epithelial cell^25^, macrophage^2^, and endothelial cells^20^. cFn contributes to support the extracellular structure framework by actively binding with cells and other matrix proteins as mentioned above.

Previous histological localization study in tooth germ revealed that Fn, which is present in the mesenchymal tissue, basement membrane, and pre-dentine, was not detected in late pre-dentine and mineralized dentine. Further, epithelial tissues of tooth germ were negative for Fn except in the stellate reticulum^12,28^. Tooth development or odontogenesis is a complex process, which needs reciprocal interaction between epithelium and mesenchyme. Various growth factors, paracrine signal molecules^27^ and extracellular matrix (ECM) proteins^6^ are believed to be essential for this process. Among those, the importance of ECM proteins is becoming increasingly apparent, since serious changes were noted in oral cancer as compared with normal tissue because of the alteration of ECM^26^. As a ubiquitous ECM, it is reported that Fn is required for calvarial osteoblast differentiation and mineralization^16^. Since Fn is expressed in early pre-dentine and disappeared in mineralized mature dentine, it is thus reasonable to conceive that Fn might play some roles in the differentiation of dental mesenchyme into dentine-forming odontoblast. Previously, Mizuno, et al.^14^ (2008) reported that Fn enhanced the osteocalcin (OCN) and osteopontin (OPN) gene expression in human dental pulp cells. However, the precise effect of Fn on cells of dental mesenchymal origin is still unclear to date. Hence, the current experiment seeks to uncover the potential interaction between Fn and odontoblast-like cells. To better imitate the real scenario that cells are surrounded by extracellular matrix proteins *in situ*, we examined the cell proliferation, differentiation and mineralization behavior in Fn-coated substrate using porcine type I collagen as a comparison ECM.

## Material and methods

### Cell culture

Rat odontoblast-like cell (MDPC-23 cell), a spontaneously immortalized cell line originally isolated from molar papillae, were cultured in Dulbecco Modified Eagle’s Medium (DMEM) (Gibco, Gaithersburg, MD, USA) supplemented with 5% fetal bovine serum (FBS) (10270-098, Gibco) at 37°C, 5% CO_2_ in a humidified atmosphere. The cells were rinsed with PBS, trypsinized (TrypLE^TM^ Express 1×, Phenol Red) (12605-010, Invitrogen, Carlsbad, CA, USA), and seeded at pre-determined concentrations. The medium was changed every other day. The cells used in this study were obtained from 25 to 33 passages.

### Protein coating on non-treated tissue culture plates

Human plasma Fn was purchased from Gibco (33016-015), reconstituted in phosphate buffered saline (PBS) (Gibco) at a stock concentration of 1 mg/mL, aliquoted, then stored at -30°C until use. Porcine skin type I collagen was obtained from Nitta gelatin (3 mg/mL, Cell matrix type I-C, 140820). Non-tissue culture grade plates (96-well plate, 351177, Falcon; 24-well plate, 1820-024, Iwaki; 12-well plate, 351143, Falcon) coated with Fn were prepared by soaking the plates in a series of concentrations (0.1, 1, 10, 20, 30, 40, 50 µg/mL, herein referred to as Fn0.1, Fn1, Fn10, Fn20, Fn30, Fn40, Fn50, respectively), collagen was coated at the concentration of 300 µg/mL (PCOL300) as recommended by manufacturer. After overnight coating, the solution was aspirated and the wells were washed twice with PBS. The cells were rinsed with PBS, trypsinized, and seeded into the 96-well plate (5×10^3^ or 1×10^4^/mL; 100 µL culture media/well), 24-well plate (5×10^3^ or 1×10^4^/mL; 1 mL culture media/well) and the 12-well plate (1×10^4^/mL; 2 mL culture media/well). The cells were cultured in DMEM supplemented with 5% FBS for the experiment. Inducer medium (10 mM β-glycerophosphate and 50 µg/mL ascorbic acid) (Wako, Osaka, Japan)^19^ was added to the culture medium from day 5. Cells seeded in wells coated with PBS served as the control.

### Cell morphology observation

Light microscopy observation: cells were inoculated at the concentration of 1×10^4^/mL. Photos of the cells were taken under light microscopy (Olympus) after 20 hours culture in 12 well plate (non-treated, 351143, Falcon).

Immunofluorescence staining: MDPC-23 cells was seeded into 24 well plate (non-treated, 1820-024, IWAKI) at the concentration of 5×10^3^/mL. Immunofluorescence staining was carried out on day 3. F-actin and nucleus were visualized by staining with Alexa Fluor 568^®^ phalloidin (A12380) (Invitrogen) and DAPI (D9542) (Sigma, St Louis, MO, USA), respectively. Briefly, cells were fixed with 4% formaldehyde (16%, methanol-free, 28906) (Thermo Fisher Scientific, Waltham, MA, USA) for 15 minutes, permeabilized with Triton-X-100 (0.1%, v/v, in PBS) (T8787-100mL) (Sigma) for 5 minutes. Alexa Fluor 568^®^ phalloidin was reconstituted in 1.5 mL methanol to generate the stock solution (200 U/mL). The stock solution was subsequently diluted (0.01 U/µL) and added into 24 well plate (200 µL/well). Tween 20 (0.05%, v/v) (Kanto Chemical) in PBS (PBST) was used to wash the cells after 1 hour incubation in room temperature. Finally, the cells were counterstained with DAPI (300 nM in PBS) for 5 minutes at room temperature and washed thoroughly by PBST. Immunofluorescence photographs were taken using EVOS^®^ FLoid^®^ Cell Imaging Station (Advanced Microscopy Group, Mill Creek, WA, USA).

### Cell proliferation assay

Cells were seeded into a 96-well plate (non-treated, 351172, Falcon) at the concentration of 5×10^3^ or 1×10^4^/mL. After incubation for 1, 2, 4, 6 days, cell counting kit-8 (CCK-8) reagent (Dojindo, Kumamoto, Japan) was added to each well to a volume of 10% (10 µL/well), followed by incubation for another 1 hour and 45 minutes at 37°C, 5% CO_2_ in a humidified atmosphere. The optical density was measured at 450 nm using a microplate reader (Bio-Rad, Hercules, CA, USA).

### Quantification of Alkaline Phosphatase (ALP) activity

Cells (1×10^4^/mL) were seeded into Fibronectin and collagen-coated 12 well plates (non-treated) and incubated for five days in DMEM supplemented with 5% FBS. On day 5, cells were removed from the culture plate using Triton-X-100 (0.1%, w/w, in distilled water) and sonicated (Bioruptor^®^, Diagenode, Seraing, Belgium) for ten minutes on ice. The lysates were centrifuged for 15 minutes at 10,483×g, 4°C (Hitachi Koki, Tokyo, Japan). The resulting supernatant was diluted and assayed for ALP activity (100-times dilution) (Wako, Osaka, Japan) and BCA protein quantification (2-times dilution) (Thermo Scientific, Rockford, IL, USA) according to the manufacturers’ instructions. Absorbance was read at 405 nm and 570 nm for the ALP assay and the protein assay, respectively.

### Quantitative reverse transcription-polymerase chain reaction (qRT-PCR)

**Figure 1:**
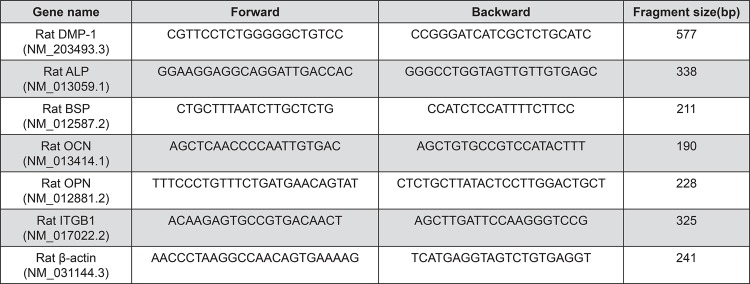
Real time RT-PCR primer

**Figure 2:**
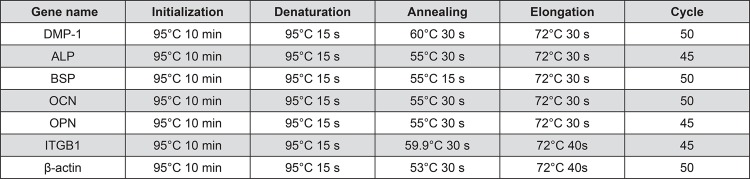
Real time RT-PCR reaction condition

Cells (1×10^4^/mL) were seeded into 12 well plates (non-treated). RNA was isolated from aliquots of cells harvested on day 7. The mRNA levels of bone sialoprotein (BSP), OCN, integrin beta 1 (ITGB1), ALP, OPN, and dentine matrix protein-1 (DMP-1) were measured using a quantitative RT-PCR machine (LightCycler^TM^ Nano, Roche, Basel, Switzerland). The mRNAs were converted to cDNA using M-MLV reverse transcriptase (Invitrogen). Real time RT-PCR was carried out in a 20 µL reaction system [cDNA: 1 µL; forward primer: 1 µL; backward primer: 1 µL; FastStart Essential DNA Green Master PCR grade H_2_O (Roche): 7 µL; FastStart Essential DNA Green Master 2× conc. (Roche): 10 µL]. The 2^-ΔΔCt^ method was used to calculate relative gene expression. The gene expression levels were normalized to β-actin mRNA level. Real time RT-PCR primer sequences and reaction conditions are listed in Figure 1 and 2, respectively. Primers were generated from Invitrogen.

### Alizarin red staining

In the same manner, cells were cultured in 12-well plates to day 10 at the concentration of 5×10^3^/mL (Low concentration) and 1×10^4^/mL (High concentration). Cells were fixed using 10% neutral buffer formalin (Wako) for 20 minutes, then the cell monolayer was stained with Alizarin red S solution (1%, w/v, in water, pH 4.0; Wako). Photographs were taken using a digital imaging system (Funakoshi, Tokyo, Japan) incorporating an inverted digital camera (Canon, Tokyo, Japan).

### Data analysis

Cell proliferation, ALP activity, and real time RT-PCR data were independently subjected to one-way analysis of variance. Once the null hypothesis of absence of differences among groups was rejected, Tukey’s multiple comparison tests were applied for pairwise comparison. A significance level of 5% was set for all analysis.

## Results

### Fn promoted MDPC-23 cells spreading and proliferation

**Figure 3:**
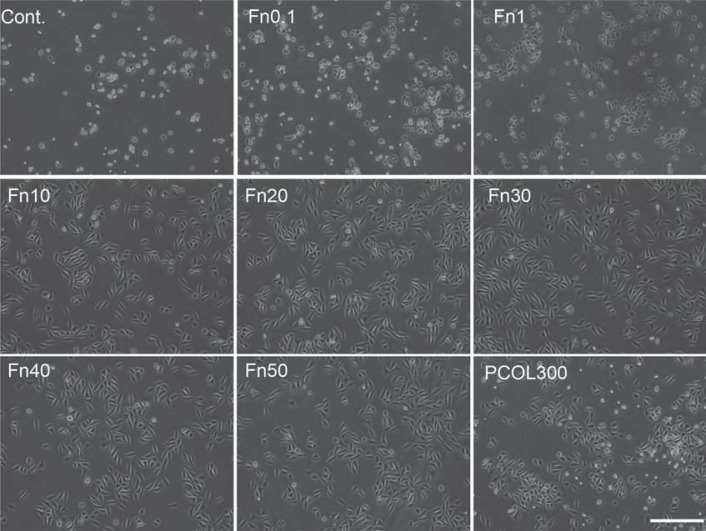
Cell morphology observation by light microscopy

**Figure 4:**
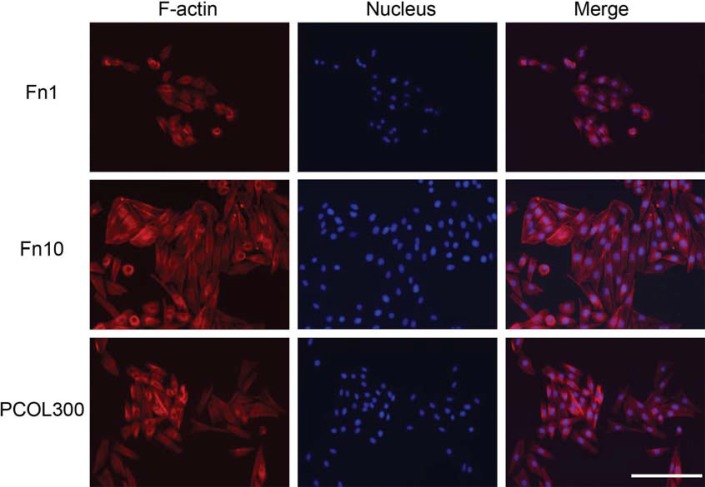
Immunofluorescence staining

**Figure 5:**
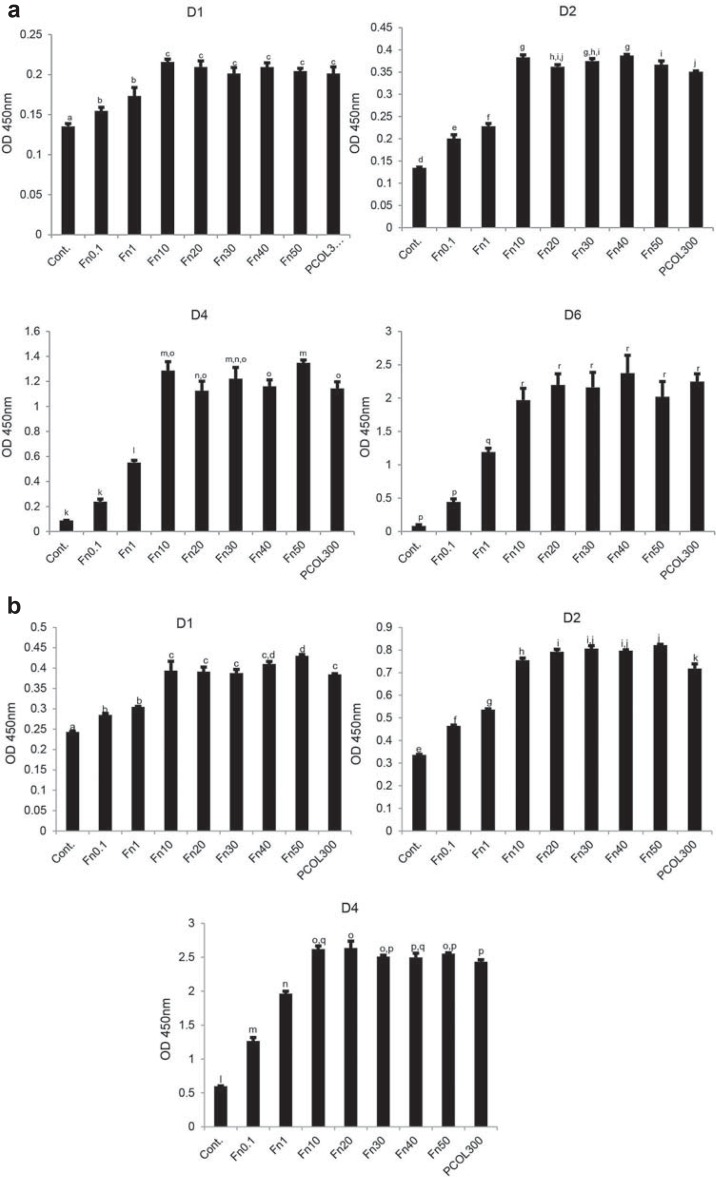
Cell proliferation activity

Photographs of light microscopy cells indicated that cells adopted well spread shape in Fn (10-50) and PCOL300 after 20 hours (Figure 3), but those cultured on control, Fn (0.1-1) were poorly spread, moreover, the attached number of cells in the three groups were much lower than Fn (10-50) and PCOL300. Given that cells growth and spreading remain comparatively unchanged in Fn with concentration over 10 µg/mL, only the immunostaining of cells grown on Fn10 was shown in Figure 4. In Figure 4, regular and parallel actin filaments are clearly observed in cells cultured on Fn (10-50) and PCOL300; however, cells assumed a polygonal or round shape while growing on Fn1. To quantify the cell proliferation activity, MDPC-23 cells were cultured in 96 well plate coated with Fn or PCOL. To further evaluate the biocompatibility of Fn, cells were inoculated at two concentrations (Low concentration: 5×10^3^/mL and high concentration: 1×10^4^/mL). Cells fail to grow in low concentration group in control well (Figure 5a); however, those cultured in Fn exhibited concentration-dependent fashion (0.1-10) of growth for the points tested four times, when the coating concentration of Fn was over 10 µg/mL, proliferation activity was maintained at a constant level; moreover, the proliferation activity in PCOL300 was quite similar to that in Fn (10-50), although significant differences exist between some Fn groups and PCOL300. In the higher concentration group (Figure 5b); cells did increase in control well, whereas the growth speed was rather low when compared with the Fn and PCOL.

MDPC-23 cells were seeded to 12 well plate (non-treated tissue culture polystyrene) at the concentration of 1×10^4^/mL in DMEM supplemented with 5% FBS. The photograph was taken 20 hours after inoculation. Cells adopted well spread and spindle shape in Fn(10-50) and PCOL-300, while those in control and Fn(0.1-1) were in round shapes. (Scale bar: 400 µm)

MDPC-23 cells in Fn(10) and PCOL-300 were well spread, F-actin formation was extensively activated in these two groups, while most of the cells grown in Fn(1) remain round in shape. (Scale bar: 200 µm)

MDPC-23 cells were seeded to 96 well plate (non-treated tissue culture polystyrene) at the concentration of 5×10^3^/mL (Figure 5a) or 1×10^4^/mL (Figure 5b) in DMEM supplemented with 5% FBS. Absorbance was read at wavelength of 450 nm. All data represents average±standard deviation (SD) from three separate experiments. Different symbols in each panel mean significant differences (p<0.05, Tukey’s test). OD: optical density. D1, D2, D4, D6 mean day 1, day 2, day 4, and day 6, respectively

### Fn increased ALP activity and the expression of odontogenic differentiation markers

**Figure 6:**
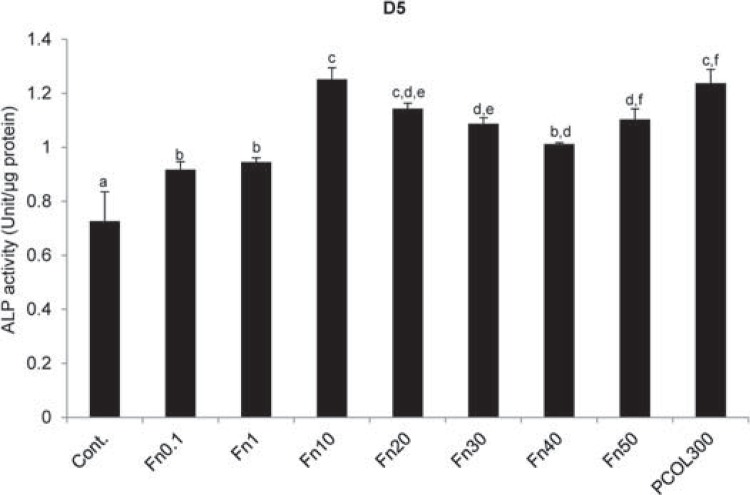
ALP activity

Alkaline phosphatase was established to be one of the phenotypic markers of odontoblastic differentiation and a critical enzyme in calcification. ALP activity was found to be the highest in Fn10 group (1.25±0.04 U/µg protein) at day 5 (Figure 6), while that of PCOL300 (1.24±0.05 U/µg protein) was almost the same as Fn10. ALP activity in Fn0.1 (0.92±0.03 U/µg protein) and Fn1 (0.95±0.02 U/µg protein) were significantly lower than that of Fn10, Fn20 (1.14±0.02 U/µg protein), Fn30 (1.09±0.02 U/µg protein), Fn50 (1.10±0.04 U/µg protein), and PCOL300.

Cells were seeded to 12 well plate (non-treated tissue culture polystyrene) at the concentration of 1×10^4^/mL in DMEM supplemented with 5% FBS. ALP activity in all the experimental groups was enhanced, in particular, Fn(10) exhibited the highest ALP activity as compared with the other groups. All data represents average±standard deviation (SD) from three separate experiments. Different symbols mean significant differences (p<0.05, Tukey test). D5 means day 5

**Figure 7:**
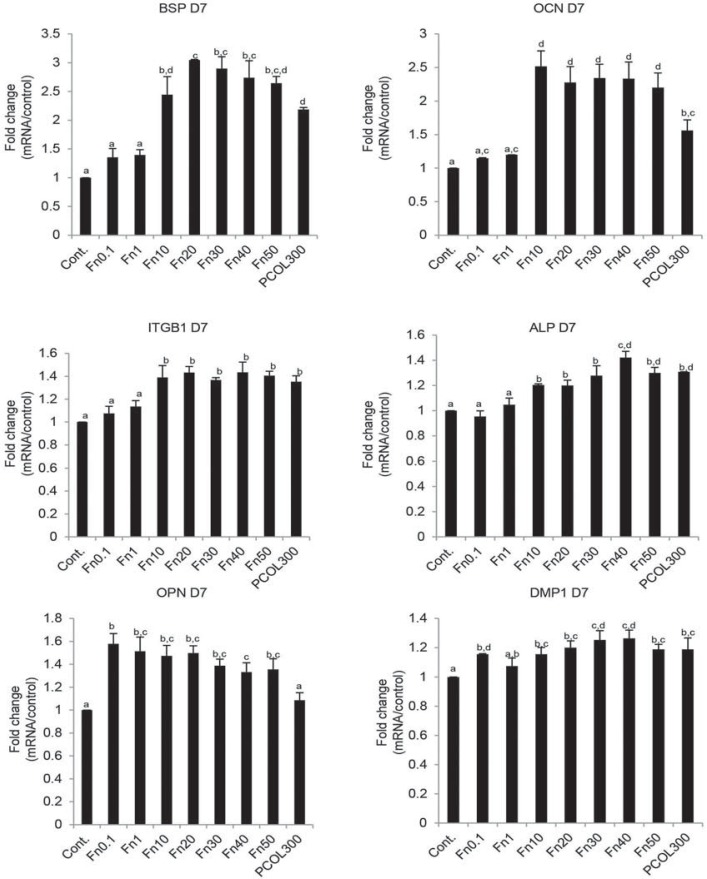
Real time RT-PCR

Similar enhancive effects were also confirmed for the mRNA expression of phenotypic markers (Figure 7). Among the markers tested, we observed that especially BSP (three fold) and OCN (2.5-fold) were significantly promoted in Fn (10-50), the fold change was also higher than PCOL300 (2.2-fold in BSP; 1.6-fold in OCN). ITGB1, ALP, and DMP1 were slightly up-regulated in Fn (10-50) and PCOL300. OPN was significantly promoted in all the coating concentration of Fn but not PCOL300.

MDPC-23 cells were seeded to 12 well plate (non-treated tissue culture polystyrene) at the concentration of 1×10^4^/mL in DMEM supplemented with 5% FBS. Odontogenesis related markers were significantly up-regulated in Fn and PCOL-300-treated substrates, especially, the expression of BSP and OCN was found to be markedly augmented in Fn(10-50). All data represent average±standard deviation (SD). Different symbols in each group mean significant differences (p<0.05, Tukey test). D7 means day 7

### Fn facilitated MDPC-23 cells calcification

**Figure 8:**
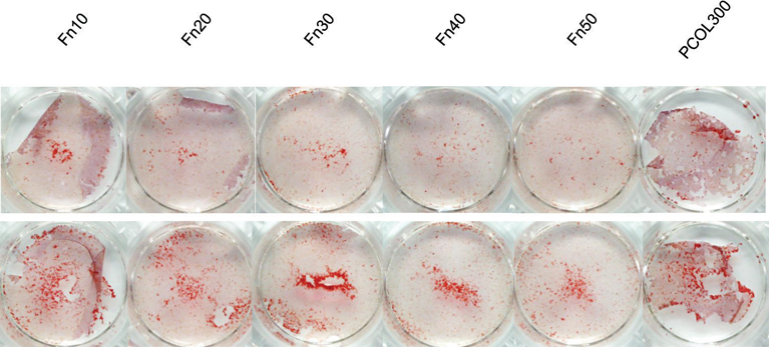
Alizarin red staining

To enhance calcification, β-GP and ascorbic acid was incorporated into culture media from 5 days. Similarly, two concentrations equal to the proliferation experiment were used for cell inoculation. After 10 days, calcifying nodules appeared in Fn (10-50) and PCOL300 in both low (Figure 8 upper) and high concentration groups (Figure 8 lower); however, higher inoculation number leads to more calcifying nodules.

Cells were inoculated in 24 well plate (non-treated tissue culture polystyrene) at the concentration of 5×10^3^/mL (upper) or 1×10^4^/mL (lower). Odontogenic reagents were added in the same manner as Figure 6, and staining was conducted at 10 days of culture. Calcification nodules were clearly observed in Fn(10-50) and PCOL300 in both low and high cell seeding density

## Discussion

Fn is an adhesive extracellular matrix glycoprotein that is involved in a variety of physiological and pathological processes. Small interfering RNA against Fn abrogated cleft formation and branching morphogenesis, while exogenous Fn facilitated and accelerated the branching and cleft formation. More importantly, Fn was found to mediate the conversion process of cell-cell adhesion to cell-matrix adhesion in human salivary epithelial cells^24^. Although Fn has been extensively studied in the development of tissues and organs, its role in the regulation of odontoblast activity remains elusive. To determine the precise role of Fn in the regulation of odontoblast activity, we evaluated proliferation and differentiation of MDPC-23 cells in Fn-coated substrates using type I collagen as a comparison with matrix protein. This study showed that culturing MDPC-23 cells in the presence of Fn can direct MDPC-23 cells down lineage of mineralizing tissue-forming cells. Specifically, key markers of odontogenic differentiation (intracellular ALP activity, phenotypic gene expression) were greater with MDPC-23 cells cultured in Fn. Furthermore, Fn facilitated calcific deposition of cells at a low seeding concentration when exposed to mineralization factors. The data presented here confirm that a regulatory relationship exists between Fn and odontoblast-like cell. The study highlighted natural cues for odontoblast differentiation within the local microenvironment *in vitro*.

Cells started to spread merely one hour after inoculation in Fn (10-50) (coated in either non-treated polystyrene or tissue culture polystyrene), which is much earlier than those grown on control (2 days) and type I collagen (18 hours) (data not shown). Actin is a family of globular proteins that form microfilaments. It is comprised of free monomer G-actin (globular) and linear polymer F-actin (filamentous). Actin is a mechanosensing tool^7^ and transduces the extracellular signals to change the shape of the cell, direct cell migration and differentiation. F-actin staining clearly showed that microfilaments formation were successfully initiated and activated on Fn10 and PCOL300, indicating that mechanosensing and signaling transduction was promoted in cells. Next, quantification of proliferation activity was conducted using CCK-8 assay. We found that Fn (0.1-50) and PCOL300 significantly increase cell proliferation as compared with the control. The activity exhibited concentration-dependent trend when concentration of Fn was lower than 10 µg/mL. It reached a state of plateau when the coating concentration of Fn was over 10 µg/mL. Further, we showed that the non-treated polystyrene was not able to support the growth of MDPC-23 cells when the cell concentration was only 5×10^3^/mL. When inoculation number increased to 1×10^4^/mL, the control did maintain a certain level of cell growth, the cell viability was markedly lower than that in Fn and PCOL300 though. The proliferation data revealed that Fn is highly biocompatible, coating of Fn to hydrophobic polystyrene facilitated cell adhesion and growth. Indeed, although Fn was not expressed in mineralized dentin and epithelial tissue in the mouse tooth germ, a strong staining of Fn using its antibody was detected between dental epithelium and mesenchyme, Thesleff, et al.^28^ (1979) hence inferred that Fn may be a potential anchorage site for cells of mesenchymal origin to differentiate into mature odontoblasts. The fluorescence staining data together with the proliferation data of this study provide a direct evidence of Fn as a potent odontoblast-like cell-adhesive, which allows MDPC-23 cells to attach, spread and proliferate rapidly.

Both calcium deposition and elevation of ALP activity were observed by Fn exposure. The results indicate that Fn promoted mineralization and is associated with increased ALP activity, since ALP can degrade β-GP and release phosphorus^1^, an early enhancement of ALP activity denotes accelerating differentiation process toward mineralizing tissue-forming cells. Besides the enhanced ALP activity, the markedly increased expression of mineralized tissue-forming genes was detected as well. BSP, OPN and DMP1 are Small Integrin Binding Ligand N-linked Glycoprotein (SIBLING) members. They possess distinct temporal and spatial expression profiles and functions. BSP is a component of mineralized tissues and is suggested to constitute approximately 8% of all non-collagenous proteins found in bone and cementum. BSP has a specific role in mediating the initial stages of connective tissue mineralization^5^ and is strongly expressed in the odontoblast-like cells of reparative dentine^11^. OPN, a highly phosphorylated glycoprotein, is important for type I collagen secretion in reparative dentine formation by newly differentiated odontoblast-like cells^23^. DMP1 is an RGD containing acidic phosphoprotein that was identified from rat incisor cDNA library; it is an inductive factor for dental pulp stem cells to differentiate into odontoblast^3^. OCN, a non-collagenous protein found in both dentin and bone, is found to be strongly expressed at 2 and 3 days post-tooth preparation, its expression correlates with reactionary dentine formation^8^. The strongest expression of OCN in Fn provides evidence of an earlier differentiation of MDPC-23 cells on the substrates. ITGB1, also known as CD29, is able to associate with a number of alpha integrin to form various types of heterogeneous integrin dimers, a recent paper suggested that interaction between fibronectin and ITGB1 is essential for ameloblast differentiation and enamel formation^22^. In particular, among the genes tested, BSP and OCN were found to be enhanced by a much higher fold change on Fn(10-50) than PCOL300, which indicated that Fn possesses a stronger capacity of initiating odontogenic differentiation as compared with PCOL300. Indeed, a previous study using dog dental pulp also confirmed that pulp cells can be stimulated to exhibit odontoblastic phenotype in response to a surface coating of plasma fibronectin even in the absence of exogenous inductive molecules^29^. The up-regulation of those mineralizing tissue markers suggested that Fn is a suitable cell expansion matrix to retain high odontogenic differentiation potential and provide a synergistic effect in the presence of odontogenic factors (β-GP and ascorbic acid) *in vitro*. Furthermore, concomitant increased expression of ITGB1 suggests that the up-regulation of odontogenic markers might be mediated, at least partially, by activation of ITGB1.

Cells secrete a wide variety of extracellular matrix that is crucial for the maintenance of normal function. ECM plays the role not only as a mechanical and structural framework, it also modulates many aspects of cell behavior, such as adhesion, migration, differentiation, and mineralization. To gain further insight into the *in vitro* effects of fibronectin and type I collagen, we have performed staining of calcified nodule. Alizarin red s was used as a vital stain for mineralization.

Fn and PCOL were pre-coated in a sterile environment in 24 well plate (non-treated polystyrene) in two different cell concentrations. Calcification of cells was induced by β-GP from 5 days. After 10 days, calcifying nodules appeared in Fn (10-50) and PCOL300-treated groups in both cell concentration groups, however, the number of nodules in groups with high number of cells was observed to be comparatively higher than that of low cell number groups. This phenomenon correlates well with previous studies using umbilical cord stem cells^31^ and human bone marrow stromal cells^13^, indicating that not only the ECM but also initial seeding density is a critical factor in determining the late stage mineralization intensity of cells. In addition, cells started to detach from control and Fn (0.1-1) at around 8 days in culture, which is the reason we do not show the alizarin staining of control and Fn (0.1-1). This has prompted us to think that cell attachment strength in Fn (10-50) is higher than that on control and Fn (0.1-1). Further detachment strength test between cells and Fn or collagen needs to be carried out.

## Conclusion

The proliferation of MDPC-23 cells cultured on polystyrene preadsorbed with Fn was promoted. Further, an early differentiation marker of odontoblast, ALP activity was significantly augmented in cells grown on Fn-treated polystyrene. The expression of odontogenesis’ markers during the differentiation and mineralization phase was increased by coated Fn. Finally, the mineralization of cells was facilitated by Fn as well. The results suggest that, a surface coating of polystyrene with Fn at the concentration of 10 µg/mL or more effectively supported the proliferation, differentiation and mineralization of odontoblast-like cells; moreover, this inductive effects of Fn was achieved at a coating concentration markedly lower than type I collagen (300 µg/mL).
